# Non-Invasive Multiphoton Imaging of Islets Transplanted Into the Pinna of the NOD Mouse Ear Reveals the Immediate Effect of Anti-CD3 Treatment in Autoimmune Diabetes

**DOI:** 10.3389/fimmu.2018.01006

**Published:** 2018-05-18

**Authors:** Robert A. Benson, Fabien Garcon, Asha Recino, John R. Ferdinand, Menna R. Clatworthy, Herman Waldmann, James M. Brewer, Klaus Okkenhaug, Anne Cooke, Paul Garside, Maja Wållberg

**Affiliations:** ^1^College of Medical, Veterinary & Life Sciences, Institute of Infection, Immunity and Inflammation, University of Glasgow, Glasgow, United Kingdom; ^2^Laboratory of Lymphocyte Signalling and Development, Babraham Institute, Cambridge, United Kingdom; ^3^Department of Pathology, University of Cambridge, Cambridge, United Kingdom; ^4^Molecular Immunity Unit, Department of Medicine, University of Cambridge, Cambridge, United Kingdom; ^5^Sir William Dunn School of Pathology, University of Oxford, Oxford, United Kingdom

**Keywords:** type 1 diabetes, islets, multiphoton, imaging, immunotherapy, anti-CD3, T cell, autoimmunity

## Abstract

We present a novel and readily accessible method facilitating cellular time-resolved imaging of transplanted pancreatic islets. Grafting of islets to the mouse ear pinna allows non-invasive, *in vivo* longitudinal imaging of events in the islets and enables improved acquisition of experimental data and use of fewer experimental animals than is possible using invasive techniques, as the same mouse can be assessed for the presence of islet infiltrating cells before and after immune intervention. We have applied this method to investigating therapeutic protection of beta cells through the well-established use of anti-CD3 injection, and have acquired unprecedented data on the nature and rapidity of the effect on the islet infiltrating T cells. We demonstrate that infusion of anti-CD3 antibody leads to immediate effects on islet infiltrating T cells in islet grafts in the pinna of the ear, and causes them to increase their speed and displacement within 20 min of infusion. This technique overcomes several technical challenges associated with intravital imaging of pancreatic immune responses and facilitates routine study of beta islet cell development, differentiation, and function in health and disease.

## Introduction

Treatment with anti-CD3 results in reversal of established diabetes in NOD mice (1) in a TGFβ-dependent manner (2), involving combination of early and long lasting mechanisms, including clearance of infiltrating cells from the islets, and a time limited reduction in the numbers of circulating T cells (3, 4). Previous work has established that anti-CD3 treatment leads to resolution of the lymphocytic infiltrates within 24–48 h (5), but it remains unclear whether this resolution is due to death of the islet-reactive cells (6) or to a change in their behavior (7–9). We have used a novel method for imaging infiltrating immune cells in islets transplanted into the pinna of the mouse ear to clarify the intra-islet events directly following anti-CD3 administration.

Disease progression in experimental type 1 diabetes is usually studied by excising the pancreata from experimental mice and analyzing the cell content, which provides a snapshot image of the inflammatory status of the islets. However, this precludes an appreciation of the dynamic temporal nature of disease processes. For studying the contribution of cellular behavior in deployment of immune effector functions, high-resolution real-time imaging must be performed (10). Multiphoton laser scanning microscopy of isolated islets from RIP-OVA mice has been used to follow the behavior of inflammatory cells infiltrating the islet. This has revealed that stable CD8^+^ T cell/APC interactions only occur early in islet invasion and are associated with induction of IFNγ production (11). Further development of intravital techniques confirmed this behavior in intact, *in situ* pancreas in the NOD mouse (12). However, the asynchronous infiltration of islets poses challenges not only for pooled islet analysis but also for longitudinal studies. Differences in disease progression between individual mice has meant that group sizes have had to be large (at least five mice per group) to estimate the effects of any treatment protocols on pancreatic events.

A way of solving the problem of continuity of observation while reducing the number of experimental mice is to visualize events in the islet through imaging, without having to remove the islet from the mouse (10). Although several groups have demonstrated feasibility of cellular imaging approaches, through surgical exposure of the pancreas (13) or an islet graft under the kidney capsule (14), the invasiveness of the procedure makes longitudinal studies impractical. Although technically challenging, insertion of an imaging window can allow visualization of islets transplanted to the kidney capsule thus facilitating repeated imaging over time (15). A major advance in permitting longitudinal studies of islet biology has been in the imaging of islets transplanted into the anterior chamber of the eye (ACE) (16), capturing images of islet vasculature (16), insulin resistance (17), beta cell mass (18, 19), as well as immune cell infiltration (19, 20). However, while this method has clear advantages for longitudinal study of islet biology, relevant immunological processes may be strongly affected by the immune privilege given by the ACE environment (21–23). Anterior chamber associated immune deviation is believed to be caused at least in part by the high levels of TGF beta in the vitreous fluid (21), and this may well affect the immune reactions we wish to record (24–26). As our interest is to monitor effects of immunomodulatory treatments, these concerns led us to examine non-invasive imaging of islets in a different site, in the pinna of the ear. The mouse ear pinna is easily accessible by various imaging modalities and compatible with studies of normal immune functions. Indeed, we have previously demonstrated longitudinal *in vivo* imaging and normal immune function (antigen drainage, lymphocyte priming, and recirculation) of lymph nodes engrafted in the pinna (27). This site has also been shown to support engraftment of a variety of different tissues including heart, thymus, kidney, adrenal gland, and spleen which not only survive, but continue to function in an organ specific way (27–30).

To test whether islets grafted into the pinna of the ear can serve as an easily accessible site for non-invasive imaging of effects of treatments aimed to decrease islet inflammation, we determined that islets can indeed become established in the pinna, and that they continue to produce insulin and glucagon therein. We then investigated whether we could use intravital imaging to further investigate the nature of the resolution of islet immune infiltration caused by administration of non-FcR binding anti-CD3 antibody, which is a mouse equivalent to Otelixizumab which has been used in clinical trials in patients (31, 32). Our data demonstrate that non-invasive imaging of islet grafts in the pinna can detect the clearance of infiltrates seen with invasive methods. Our results also show that in addition to any cell death occurring after injection of anti-CD3, there is an immediate increase in T cell motility after administration, indicating reduced interaction with targets and APC which can have profound effects on T cell tolerance (33, 34).

This technique enables immunological processes to be followed in individual animals over several time points with minimal discomfort, using fewer laboratory animals and allowing for more accurate data acquisition.

## Materials and Methods

### Mice and Diabetes Detection

NOD, NOD-*scid* NOD, NOD-hCD2-GFP (35), Kaede-C57BL/6 (36) and NOD Foxp3-GFP mice (37), hCD2-GFP (38), and CD11cYFP (39) were bred and maintained under specific pathogen-free barrier conditions. C57BL/6 mice were purchased from Charles River. This study was carried out in accordance with the recommendations of the Animals (Scientific Procedures) Act. The protocols were approved by the UK Home Office and performed under the Project Licences of PG, KO, and MW. Mice were housed in individually ventilated cages with free access to standard chow and water. The facility is kept on a 12 h light, 12 h dark cycle. The humane endpoints for these experiments specify that any mouse that loses more than 15% of its body weight (compared with healthy littermates), or in other ways looks unwell and likely to exceed the Home Office standard of moderate severity must be culled. However, no mice used in this study required early culling. At the end of the experiments, mice were culled using a CO_2_-chamber followed by dislocation of the neck. In cases where we were harvesting islets for transplantation it was important to maintain an intact bile duct, and death was instead confirmed through palpation of the chest to assess the absence of a heart beat followed by confirmation of cessation of blood flow. Diabetes was detected using Diastix reagent strips (Bayer Diagnostics, Basingstoke, UK) and confirmed by a blood glucose measurement of >13.3 mM, using a Breeze2 blood glucose meter (Bayer). All animal work was conducted under UK Home Office project licence regulations after approval by the Ethical Review Committees of the University of Cambridge and Glasgow, respectively.

### Islet Isolation and Transplantation

Pancreatic islets were isolated through inflation of the pancreas *via* the bile duct (40), and islets were prepared for transplantation by insertion into polyethylene tubing as previously described (41). Islets were prepared from mice ranging from 6 to 14 weeks of age, and recipient mice were between 10 and 12 weeks of age. Analgesic (Temgesic, Reckitt Benckiser) was administered s.c. in the scruff prior to anesthesia (Isoflurane, Abbott Laboratories). Ears were immobilized using double sided sticky tape for creation of a pocket on the ventral side of the pinna using forceps. A single piece of tubing containing the islets was inserted into the pocket, and approximately 50 islets deposited into the space. The incision was then closed with veterinary-grade glue (Vetbond, 3M).

### Aglycosyl Anti-CD3 Antibody

The non-Fc receptor binding anti-mouse CD3 antibody (agly anti-CD3) was generated through genetic engineering in the Waldmann Laboratory, University of Oxford as previously described (9). The antibody that recognizes human CD3 (Otelixizumab) was used as a non-binding isotype control.

### Multiphoton Laser Scanning Microscopy

Intravital microscopy studies were carried out using a Zeiss LSM7 MP system or a Leica SP8 MP system (Molecular Immunology Unit, University of Cambridge) equipped with a tuneable Titanium: sapphire solid-state two-photon excitation source (4W, Chameleon Ultra II, Coherent Laser Group) coupled to an Optical Parametric Oscillator (Chameleon Compact OPO; Coherent). Movies were acquired for 20–30 min with an X–Y pixel resolution of 512 × 512 in 2 µm Z increments. 3D tracking was performed using Volocity 6.1.1 (Perkin Elmer, Cambridge, UK) or Imaris (Bitplane). Cells were identified using intensity thresholding and object volume. Track plots are included to demonstrate the actual migration of cells relative to their point of origin. To calculate displacement rate, we first calculated the straight line distance between the point of origin and the point of termination of a cell track and then divided the distance by the duration of the cell track in minutes. Maximum speed is presented in micrometer per minutes. Meandering index (also known as confinement ratio or chemotactic index) is a ratio defining track straightness (ratio of displacement of the cell to the total length of the track) where 0 represents a highly confined cell that returns to its starting position, and 1 being a cell traveling in a completely straight line. Mice were anaesthetized using Domitor (50 mg/kg Ketamine and 0.5 mg/kg Medetomidine i.p.) and placed on a heated stage. The ear of interest was mounted on a purpose-built stand with the use of veterinary-grade glue (Vetbond; 3M), enabling warming of tissue throughout the experiment (maintained at 37°C). To allow repeated imaging sessions, veterinary glue was dissolved with 70% ethanol, and mice given Antisedan (1 mg/kg Atipamezole s.c.) for reversal of the Domitor anesthesia. Blood vessels were visualized by i.v. injection of non-targeted quantum dots (Qtracker 655, Life Technologies). SYTOX was used to image cell death *in situ*. 10 µl of 10 µM SYTOX orange (Molecular Probes, Thermo Fischer, UK) was injected into the ear pinna 10 min prior to imaging to allow visualization of dead cells (42). To image naïve T cells in lymph nodes, DsRed expressing CD4^+^T cells from unimmunized mice were magnetically sorted from hCD2-DsRed animals using mouse CD4^+^T cell isolation kits (Miltenyi Biotec, Surrey, UK). 2–3 × 10^6^ purified T cells were then adoptively transferred into naïve CD11cYFP recipient mice. Popliteal LNs were excised 24 h later for imaging. LNs were maintained by being continuously bathed in warmed (37°C), oxygenated CO_2_ independent medium (43). For studies of the effects of aglycosylated anti-CD3, 20 µg of active antibody or isotype control (9) was injected i.v. as indicated. Imaging was interrupted for injection of antibody, and started no more than 5 min after injection. The results achieved in the multiphoton imaging facility at the University of Glasgow were also replicated in the facility at the Babraham Institute, Cambridge. A 1:1 mixture of Hypnorm (25 mg/kg) and Hypnovel (12.5 mg/kg) injected i.p. was used to induce anesthesia during imaging at the Babraham Institute. Movies were acquired for the indicated time with an X–Y pixel resolution of 512 × 512 in 6.23 µm Z increments and a total depth of 62.3–68.53 µm with a 30 s frame interval.

For imaging of islets in the pancreas at the University of Cambridge, mice were anesthetized using 3% isoflurane, the pancreas externalized, submerged in surgical saline solution, and immobilized under a cover slip and the mouse placed in a 37°C environmental chamber for imaging. Islets were imaged using a 20× water immersion objective for up to 1 h per movie. Movies were acquired with an X–Y pixel resolution of 512 × 512 in 2 µm Z increments and a total depth of 54–67 µm with a 40 s frame interval.

### Immunofluorescence

Pancreata and graft-bearing pinnae were fixed in 4% PFA for 72 h, dehydrated in sucrose, and mounted in OCT. 10 µm sections were cut on a Leica cryostat and placed on Polysine slides (VWR), air dried, and fixed again for 10 min in acetone. Sections were stained using guinea pig-anti-insulin antibody (DAKO) at a 1:40 dilution and detected with anti-guinea pig Alexa 546 (Molecular Probes). Rabbit anti-human glucagon (Millipore) was used at a 1:50 dilution and detected with anti-rabbit Alexa 488 (Molecular Probes). Anti-mouse CD4 (BD) was detected with anti-rat Alexa 488 (Molecular Probes). Nuclei were visualized with DAPI (Molecular Probes). The sections were viewed using either a Zeiss Axioskop 2 or LSM700 Confocal (Zeiss) and processed using Zen software.

### Flow Cytometry

Cells were prepared from pancreas and islet grafts in pinna through manual dispersion of the tissue followed by digestion for 10 min at 37°C in 0.5% collagenase P solution (Sigma). Cell suspensions from lymph nodes were prepared through dispersion between glass slides. All cell preparations were resuspended in FACS buffer (PBS with 0.5% BSA), filtered through 30 µm Celltrics filters (Partec), incubated with Fc-block (eBioscience) and then stained with CD19 Alexa647 (eBioscience), washed, and resuspended in PBS containing 5% 7AAD (BD Bioscience). Data were collected on a Cyan Cell Cytometer (DAKO) and the data analyzed using FlowJo (Tree Star Inc.).

### Statistical Analysis

Differences between two groups were assessed with a non-parametric Mann–Whitney test, and for multiple comparisons we used the non-parametric Kruskal–Wallis test with a Dunn’s post-test.

## Results

### The Pinna of the Ear as a Site for Islet Transplantation

**Figure 1:**
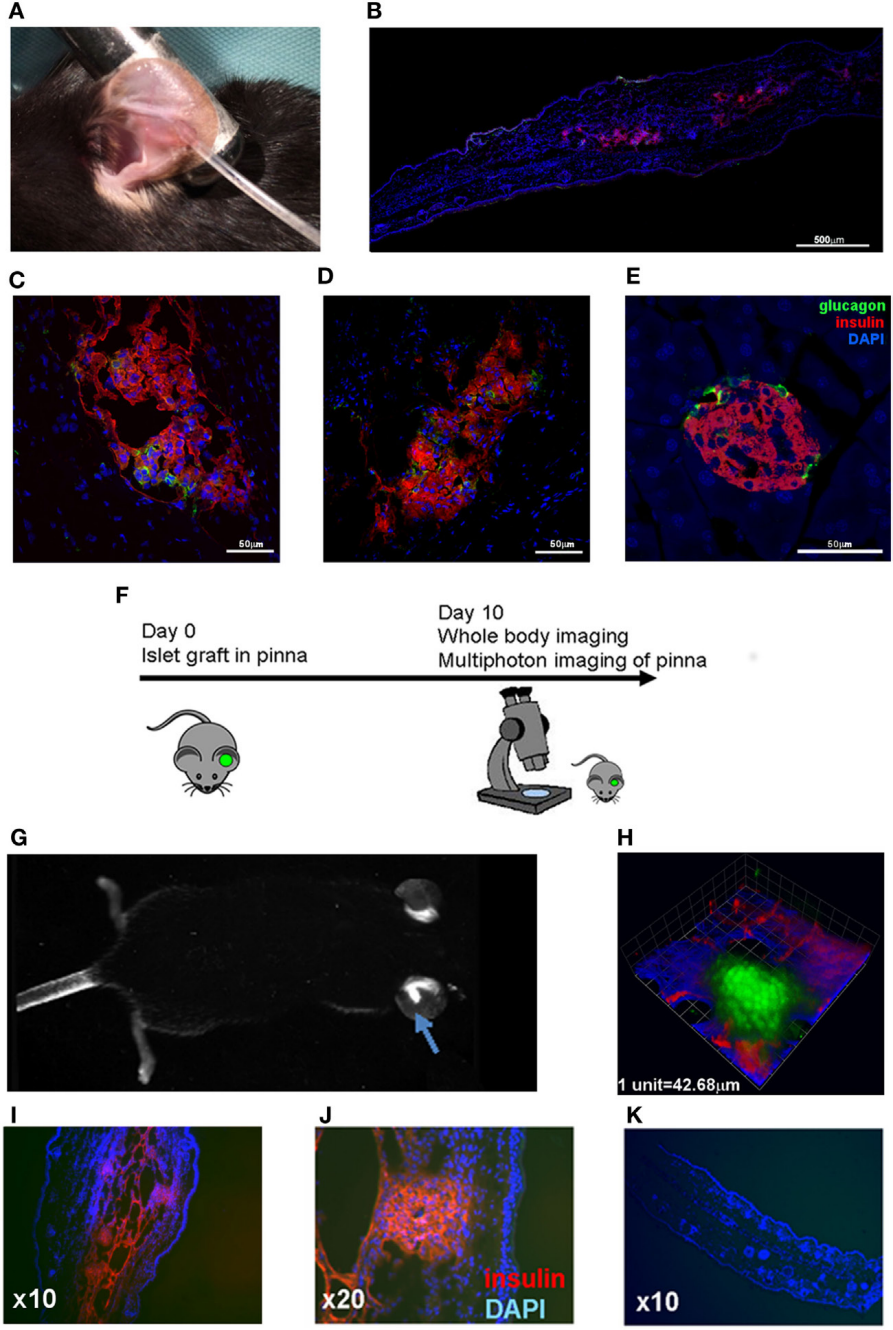
Pancreatic islets grafted into the pinna of the ear retain capacity to produce insulin and can be used for non-invasive imaging. **(A)** C57BL/6 islets were grafted into the pinna of syngeneic C57BL/6 recipients. **(B)** 12 weeks later, islets were readily detected in the pinna, and higher magnification revealed glucagon positive cells (green) among the insulin positive beta cells (red) **(C,D)**. The same staining was performed on islets in the pancreas of the graft recipient **(E)**. **(F)** Islets isolated from a Kaede transgenic mice were grafted into the pinna of syngeneic C57BL/6 mice. On day 10, the graft recipient was anaesthetized and analyzed with whole body imaging and multiphoton microscopy. **(G)** On day 10, green fluorescent islets could be detected in the pinna using whole body imaging. An arrow indicates the fluorescent mass in the grafted ear. **(H)** The green fluorescent grafted islets could also be detected with multiphoton imaging (3D rendering of a Z-stack capture). **(I,J)** The graft bearing pinnae were fixed and stained to check for insulin production (red), and compared with a cross section of a control pinna **(K)**. The selected images are representative of results from groups containing at least three mice.

To demonstrate the feasibility of the pinna as a suitable graft site for islets, we grafted C57BL/6 islets into the pinna of syngeneic C57BL/6 recipient mice. These mice have no pre-existing autoimmunity to islets, and can accept syngeneic islet grafts under the kidney capsule (44). Islets of Langerhans were isolated (40) and prepared for transplantation as previously described (41). The islets were then deposited into a small pocket in the skin on the ventral side of the pinna (Figure 1A). Grafted islets could be detected in the ear pinna up to 12 weeks post-transplantation and were seen to produce insulin (Figure 1B) (later time points were not assessed). Indeed, insulin and glucagon producing cells were readily detected in the islets in the pinna (Figures 1C,D). Although the gross architecture of the islets grafted into the pinna appeared normal, glucagon-producing alpha cells were found throughout the islet rather than along the perimeter edge as seen in islets directly from the pancreas (Figure 1E).

To determine whether the pinna would be feasible for non-invasive imaging, we transplanted fluorescent islets from Kaede C57BL/6 mice (36) into C57BL/6 recipients (Figure 1F). The fluorescent islets were accepted, and fluorescence could be detected using whole body imaging (Figure 1G) and multiphoton imaging (Figure 1H; Movie S1 in Supplementary Material). The grafted pinnae were removed after imaging and stained for insulin, again demonstrating the presence of insulin-producing cells in the grafted islets (Figures 1I–K). Thus, islets grafted to the ear pinna can survive, are functional, and readily amenable to non-invasive imaging.

### Use of Islet Grafts in the Pinna to Assess Immune Infiltration

**Figure 2:**
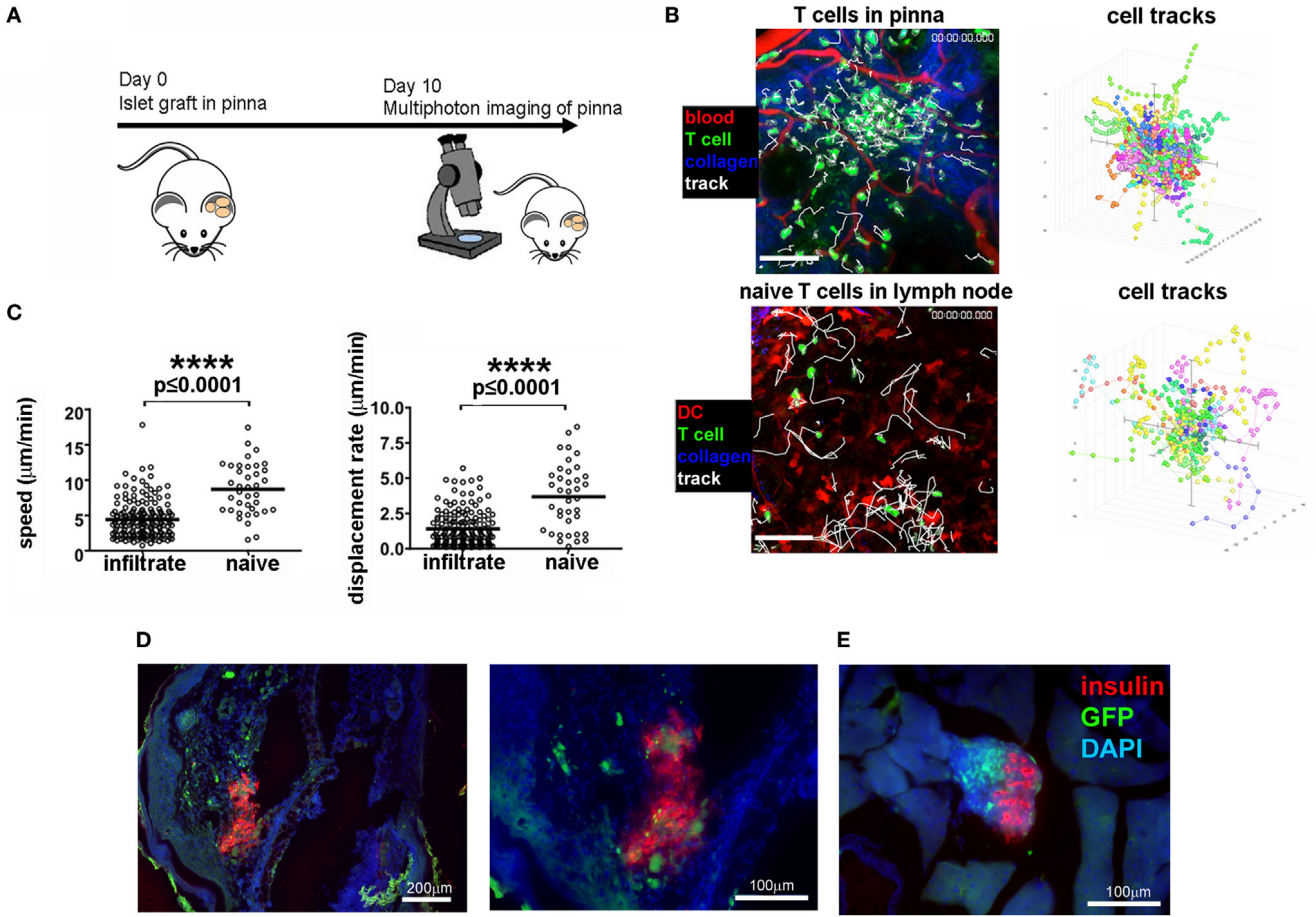
Imaging of T cell infiltration in NOD-*scid* islets transplanted into the pinna of the ear of a CD2-GFP NOD recipient. **(A)** On day 0, islets were isolated from a NOD-*scid* mouse and grafted into the pinna of a CD2-GFP NOD mouse. On day 10, the graft recipient was anesthetized and the graft imaged using multiphoton microscopy. **(B)** Multiphoton imaging showing either islet-infiltrating GFP^+^ T cells (green) around a grafted islet in a 12-week old NOD recipient intact ear pinna (top panels), or naïve T cells moving in a lymph node (bottom panels). Scale bars represent 100 µm. The right-hand side flower plots demonstrate the movement of the T cells during imaging **(B)**, presented in graph form in **(C)**. After imaging, the pinna **(D)** and pancreas **(E)** were fixed and stained for insulin (red) and DNA stain DAPI (blue). T cells can be identified by green GFP signal. The selected images are representative of results from groups containing ≥ three mice.

Given that the grafted tissue can be imaged non-invasively, we sought to determine the potential for monitoring the T cell infiltrate that mediates β cell destruction in NOD mice. We grafted NOD-*scid* islets into the pinna of CD2-GFP NOD recipients (Figure 2A), allowing tracking of T cell responses where there is existing immune reactivity to islet antigens and established pancreatic immune infiltration. Ten days after transplantation, foci of GFP fluorescence could be detected, as well as individual immune cells in the infiltrate whose movements could be tracked (Figure 2B, top panel; Movie S2 in Supplementary Material). Imaging of naïve T cells in lymph node (Figure 2B, lower panel) demonstrates that the cells infiltrating the transplanted islets behave more like activated cells engaging a target (45), displaying a lower velocity and displacement than naive cells (Figure 2B, plotted in Figure 2C). Insulin staining of the islet graft after fixation of the pinna demonstrates the presence of insulin producing cells in the pinna, with considerable presence of infiltrating immune cells (Figure 2D), which corresponds to the presence of advanced infiltration of the islets in the pancreas of the same graft recipient (Figure 2E, right panel). Using an injectable cell death dye, Sytox, we could also monitor cell death in the grafted pinna while imaging (Movie S3 in Supplementary Material). Use of a different transgenic recipient strain in which all Foxp3 expressing cells also express GFP, enabled imaging of the presence and behavior of Foxp3^+^ Treg cells in the grafts (Movie S4 in Supplementary Material). These data demonstrate that infiltration of lymphocytes into the islet graft can be monitored and quantified *in vivo* without need of surgical exposure of the tissue for multiphoton imaging.

### Determination of Effects of Aglycosyl Anti-CD3 Treatment on Islet Grafts in the Pinna

**Figure 3:**
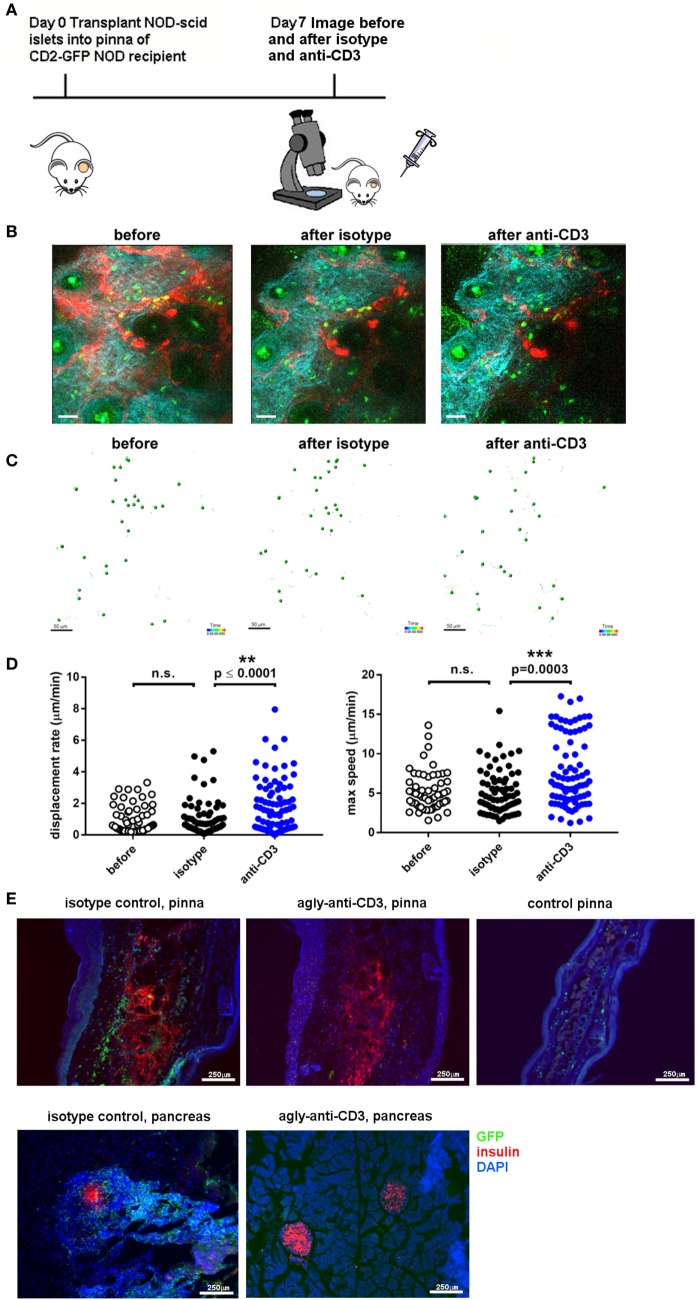
Imaging of T cell infiltration in NOD-*scid* islets transplanted into the pinna of the ear of a CD2-GFP NOD recipient before and after administration of agly anti-CD3. **(A)** On day 0, islets were isolated from a NOD-*scid* mouse and grafted into the pinna of a CD2-GFP NOD mouse. On day 7, the graft recipient was anesthetized and the graft imaged using multiphoton microscopy. Then 20 µg of first isotype control antibody and then agly anti-CD3 was administered i.v. and the graft imaged again. **(B)** Still images from acquired Z-stack longitudinal multiphoton imaging showing islet-infiltrating GFP^+^ T cells (green) moving around a grafted islet in a 12-week-old NOD recipient before injection [**(B)**, left panel], after injection of isotype control antibody [**(B)**, center panel], and after injection of aglycosyl anti-CD3 [**(B)**, right panel]. The white bar represents 50 µm. Red = blood vessels, blue = collagen (secondary harmonic signal), green = GFP. **(C)** Depiction of the individual tracks of infiltrating cells during the imaging period in the indicated conditions. **(D)** Plotting of the recorded displacement rate (left) and max speed (right) of islet infiltrating cells before and after administration of isotype antibody or aglycosyl anti-CD3. The data are representative of five independent experiments and differences between groups were analyzed using a non-parametric Kruskal–Wallis test with a Dunn’s post-test for multiple comparisons. **(E)** Section staining of pinnae [**(E)**, top panels] and pancreas [**(E)**, bottom panels] from isotype control injected graft recipients [**(E)**, left-hand panels] and aglycosyl anti-CD3 injected [**(E)**, middle panels] or control pinna (top right panel). Red = insulin, blue = DAPI, green = GFP.

**Figure 4:**
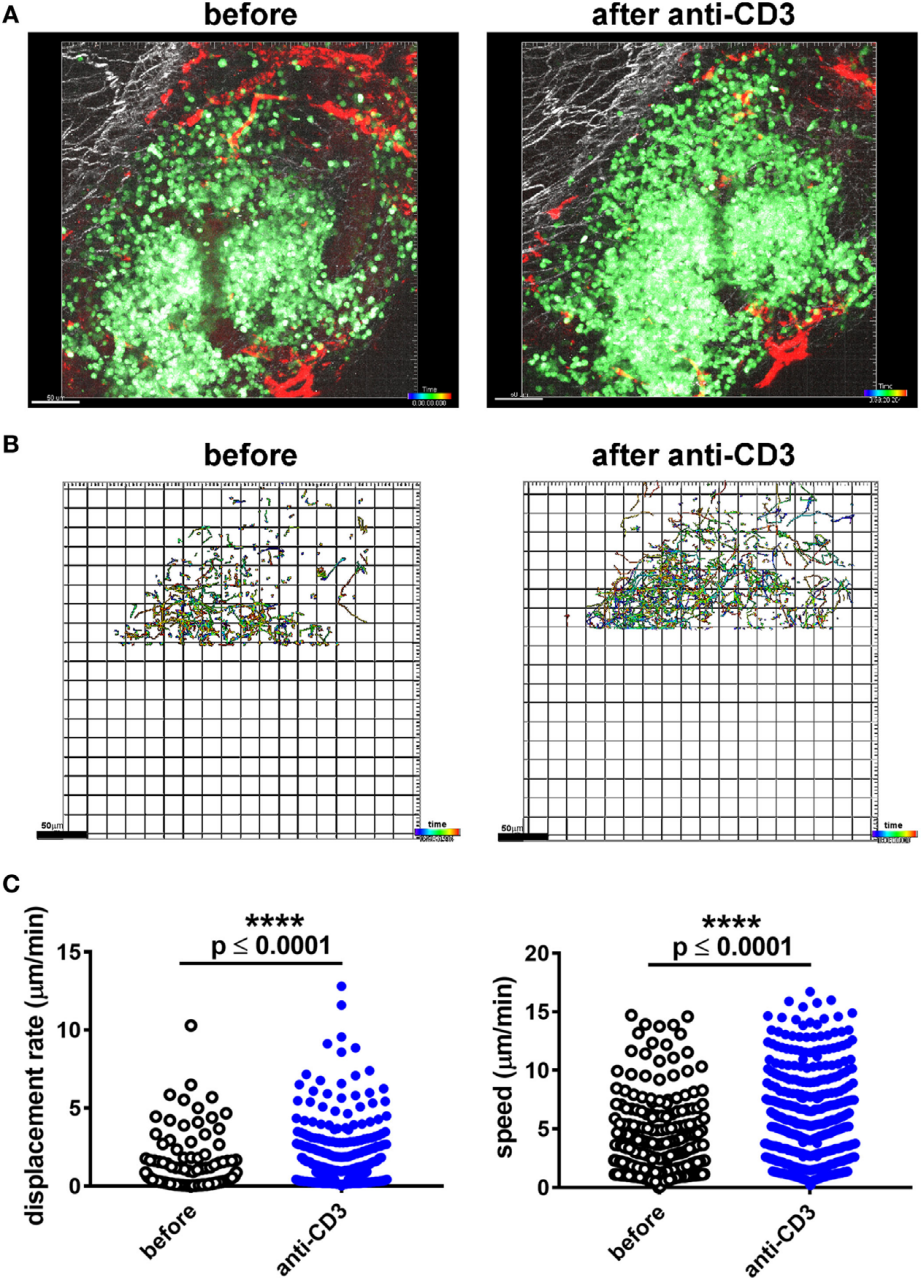
Imaging of T cell infiltration in a pancreatic CD2-GFP NOD islet before and after administration of agly anti-CD3. **(A)** The mouse was anesthetised, the pancreas externalized, and mounted for multiphoton microscopy imaging. The infiltrated islet was imaged before and after i.v. administration of 20 µg of agly anti-CD3. Still image from acquired Z-stack longitudinal multiphoton imaging showing islet-infiltrating GFP^+^ T cells (green) moving around an islet in a 12-week old NOD recipient before injection [**(A)**, left panel], and after injection of aglycosyl anti-CD3 [**(A)**, right panel]. The white bar represents 50 µm. Red = blood vessels, blue = collagen (secondary harmonic signal), green = GFP. **(B)** Depiction of the individual tracks of infiltrating cells during the imaging period (59 min and 20 s) in the indicated conditions. Data were only acquired from the top part of the capture, as the infiltrates in the lower parts were too dense to track. **(C)** Plotting of the recorded displacement (left) and speed (right) of islet infiltrating cells before and after administration of aglycosyl anti-CD3. The difference between groups was assessed using a non-parametric Mann–Whitney test, and the data are representative of three independent experiments.

To assess whether immune infiltrate into islets grafted into the pinna reacted in a similar way to established immunotherapy as infiltrates in the pancreas, we assessed the response to injection of a non-lytic aglycosyl anti-CD3, which can prevent rejection of allografts (46) and reverse overt diabetes through loss of islet infiltrating cells (1, 6). We grafted NOD-*scid* islets into the pinna of CD2-GFP NOD mice on day 0, and imaged infiltration on day 7 (Figure 3A). After an initial period of imaging (Figure 3B, left hand panel; Movie S5 in Supplementary Material), first 20 µg of isotype antibody was injected i.v. (Figure 3B, middle panel; Movie S6 in Supplementary Material) and then 20 µg of agly CD3 was injected i.v., and the same area of the graft imaged again to monitor any change in the behavior of infiltrating cells (Figure 3B, right-hand panel; Movie S7 in Supplementary Material). Imaging was interrupted for injection of antibody and started no more than 5 min after injection. Analysis of the movement of the cells before and after injection of agly CD3 (Figure 3C) demonstrated a marked increase in speed and displacement rate after the injection of anti-CD3 but not isotype antibody, indicating that the active antibody had an immediate effect on the islet reversing the T cell arrest required for TCR binding and T cell activation (Figure 3D) (47). The injection of isotype antibody induced modest increase in cell displacement in some experiments, but not to the same extent as the active anti-CD3 antibody (Figure 3C). The observed increase in T cell speed and displacement was confirmed by identical results recorded when performing the procedure and imaging in the facilities at the University of Glasgow (Figure S1 and Movies S8 and S9 in Supplementary Material). When we injected 20 µg of agly CD3 on days 7, 8, and 9, and imaged again on day 10, we found that the infiltrates in the treated mouse, which had abundant infiltrates on day 7, were dispersed after 3 days of agly anti-CD3 treatment (Figure S2 in Supplementary Material), mirroring the effect of this treatment regimen in islets in the pancreas. Staining of sections of grafted pinnae and pancreata of islet graft recipient mice either receiving agly anti-CD3 (Figure 3E, central panels) or not (Figure 3E, left-hand panels), also demonstrated the reduction of infiltrating T cells both in islets in the pinna (Figure 3E, top panels) and pancreas (Figure 3E, bottom panels) after treatment. These results were further supported by assessment of infiltrating cells *via* flow cytometry of excised grafts from the pinna (Figure S2 in Supplementary Material), showing a decrease in the presence of GFP positive T cells in mice treated with agly anti-CD3 compared with isotype antibody treated controls. We confirmed that the observed change in behavior in the islet infiltrating cells in the pinna is representative of the events in the native pancreas after injection of agly anti-CD3 by direct imaging of the pancreas. We found that islet infiltrating T cells increase their speed and displacement after injection of agly anti-CD3 antibody in a similar way as the infiltrates in the pinna islet grafts (Figure 4; Movies S10 and S11 in Supplementary Material).

## Discussion

By engrafting islets to the ear pinna, we have circumvented issues of immune privilege and invasiveness to allow study of immune mechanisms mediating beta cell destruction and therapeutic efficacy. It has been shown that the pinna of the ear is a suitable site for transplantation of a range of tissues (28–30) and for multiphoton imaging (27), supporting conventional lymphatic drainage, revascularization of the grafted tissue, and normal lymphocyte responses (27, 28). Furthermore, the pinna represents an easily accessible tissue for the grafting procedure as well as for microscope access and minimization of drift during imaging, making this an ideal site for longitudinal *in vivo* cellular imaging studies. We show that anti-CD3 treatment leads to an immediate increase in movement and displacement of infiltrating immune cells in islets in the pancreas, and that we can use imaging of islets transplanted into the pinna to image this process in a less invasive way. We present unprecedented data on the nature and rapidity of the effect on the islet infiltrating T cells.

In the more than 20 years since Lucienne Chatenoud’s pioneering experiments demonstrating reversal of diabetes in experimental mice (1), the mechanisms through which the anti-CD3 antibodies exert their effect have been gradually clarified. They work through a wide array immediate and long-term effects, including downregulation of the CD3 signaling complex (48), induction of anergy and apoptosis (49, 50), and increased regulation through elevated levels of TGF beta (24) and an expanded Treg pool (6, 51), as reviewed in Ref. (3, 4). A compelling suggestion is that the observed increase in the Foxp3^+^ Treg population after anti-CD3 treatment is due to a preferential depletion of activated T cells (6), a theory which has been supported by studies using OVA specific cells (6, 46). Our studies utilizing GFP labeled islet antigen-specific Th1 cells for tracking cell fate, while replicating the finding of a retained Treg pool, did not, in this model, find a preferential depletion of islet-specific effector T cells by anti-CD3 treatment (9). Instead we found an induction of anergy accompanied by an increase in PD-1 expression in this cell subset, which prevented further activation of islet antigen-presenting APC. Induced anergy in effector T cells is often referred to as exhaustion (52) as it shares many of the same markers, importantly high expression of PD-1. There is an accumulation of CD8^+^ T cells with an exhausted phenotype in type 1 diabetes patients that respond well to Teplizumab treatment (as measured through preservation of c-peptide production) (53, 54). Studies in islet allograft models also indicate induction of anergy and upregulation of PD-1 on both graft infiltrating CD8^+^ T cells (55) and CD4^+^ T cells (8) after anti-CD3 treatment, suggesting that induction of anergy in the effector population rather than expansion of the Treg pool is what affords long-term tolerance after anti-CD3 treatment (8). The requirement for effects on already primed cells is also supported by studies of the importance of timing of the treatment. Anti-CD3 treatment is only effective if administered once the mice have become diabetic, and not before (5). The effects of anti-CD3 treatment on tolerance to transplanted tissue is also highly dependent on the timing of administration (46, 56), with treatment coinciding with the priming of allo-reactive immune responses (3–7 days after transplant) affording long-term graft survival due to preferential activation of tolerogenic pathways (7).

It is well established that recently diagnosed diabetic NOD mice revert to euglycemia within 24–48 h after anti-CD3 treatment (5). We have used non-invasive imaging of islets transplanted into the pinna of the ear to assess just how quickly the effects of the anti-CD3 treatment can be observed. Autoimmune infiltration of islets grafted to the ear pinna is readily imaged directly through the skin allowing both dynamic and longitudinal imaging of lymphocyte responses. We observed that the T cells infiltrating the transplanted islets were less mobile than those swarming around in a naïve lymph node, most likely indicating that they had received activation through the TCR and were engaged with a target (57). This mirrors the behavior observed in tumor infiltrating T cells, which arrest on tumor cells expressing their cognate antigen (58, 59), the antigen-induced migration arrest of T cells infiltrating a mis-matched skin graft (60) and mycobacteria-specific T cells in a cognate antigen rich environment (61). When we administered the non-FcR binding anti-CD3 antibody, we saw a rapid increase in the mobility of the infiltrating T cells in the islets, more similar to the swarming behavior of T cells which have not yet found a suitable APC. A cessation of the TCR-MHC interaction, be it either CTL attacking a beta cell or a CD4^+^ T cell receiving activating signals from an intra-islet APC, will naturally have dampening effects on the ongoing inflammation. As CTL function as “serial killers,” moving from one target to the next (62), an interruption of the progress of just one cell can save several potential targets. Interaction time is also of crucial importance for acquisition of effector properties (43), and decreased interaction time between APC and T cell contributes to the tolerization of both CD8^+^ T cells (33) and CD4^+^ T cells (34), reducing their production of proinflammatory cytokines and expression of surface activation markers. The long-lasting effects of short-term anti-CD3 treatment involve the interruption and qualitative change of the anti-islet immune response, and non-specific bystander T cells are cleared from the islet just as the islet-specific effectors are. This indicates that anti-CD3 treatment affects not only the T cells, but by extension also the pro-inflammatory milieu in the islet, possibly *via* effects on antigen-presenting cells (9), breaking the cascade of proinflammatory events that would otherwise result in diabetes. Thus, the observed increase in motility may well influence both the short-term and long-term effects of anti-CD3 treatment, i.e., the cessation of interaction and downregulation of CD3, but also a skewing of the ensuing immune response to be more tolerogenic. In relation to the demonstrated need for TGFβ, we surmise that the consequent clearance of residual apoptotic cells during the resolution phase of healing may trigger release of active TGF-β, modifying the local microenvironment to acquire a level of immune privilege (63). In summary, imaging revealed a hitherto undescribed rapidity in response to treatment, with graft infiltrating cells recovering motility and thus terminating the cell–cell interactions required for T cell activation (47) within 20 min of injection of the aglycosyl anti-CD3 antibody. Repeated imaging of the same animal revealed the eventual loss of infiltrating T cells at the graft site.

Importantly, this technique offers an opportunity for reduction of the number of experimental animals used in research, as the non-invasive nature of the investigation allows longitudinal monitoring of the same islets in the same host. In addition, current models employing grafting of islets require a high degree of surgical proficiency while insertion into the ear pinna is minimally invasive. Traditional use of large groups of mice for temporal studies introduces experimental variation due to differing levels of immune infiltration in the pancreatic islet. The ability to collect data at several time points in the same experimental mouse negates this variance and enhances data quality. The amenable nature of beta islet grafting to the ear pinna makes this technique a new tool for studies of immune response in diabetes. The development of functional probes and new analytical tools is increasing the options for investigating immune responses *in vivo* with multiphoton imaging, and offers exciting opportunities for studies of type 1 diabetes. Combined use of IFN-γ reporter mice and Foxp3 reporter mice could clarify any differences in behavior of different cell subsets in the islets in response to treatment, and recent advances may soon allow studies of molecular dynamics in individual islet infiltrating cells (64). We have used islets transplanted into the pinna to clarify the events immediately following anti-CD3 treatment, and we now look forward to investigating the parameters required for establishment and maintenance of long-term tolerance. The use of the pinna as a site for islet transplantation could also facilitate studies of changes in islet mass over time and beta cell physiology (65, 66), and we hope that a combination of advances in both beta cell and immune cell imaging will lead to new insights into the biology of type 1 diabetes and thus, opportunities for treatment.

## Ethics Statement

This study was carried out in accordance with the recommendations of the Animals (Scientific Procedures) Act. The protocols were approved by the UK Home Office and performed under the Project Licences of PG, KO, MC, and MW.

## Author Contributions

MW, RB, AR, JF, and FG performed the experiments. RB, MW, PG, FG, KO, JF, MC, and AC planned the experiments. MW wrote the manuscript, RB, JB, PG, HW, AR, FG, KO, JF, MC, and AC contributed to discussion and reviewed/edited manuscript.

## Conflict of Interest Statement

The authors declare that the research was conducted in the absence of any commercial or financial relationships that could be construed as a potential conflict of interest.

## Supplementary Material

Movie S1 Kaede-C57BL/6 fluorescent islets transplanted into the pinna of the ear of a wt C57BL/6 mouse. Image capture starts at the bottom of the Z stack (middle of the ear) and moves to the top (up through the dermis to the epidermis). The movie shows the Kaede transplanted islets in green, vasculature in red (Qdot tracer given i.v. prior to imaging), and the blue is second harmonic autofluorescence generated by the dermal collagen fibers. Each stack is roughly 150 µm thick. Loops of blood vessels going around hair follicles (which are the round holes in the collagen network) show up in red.

Movie S2 16-min capture of pinna with NOD scid islets transplanted into a NOD-hCD2-GFP recipient. T cells are green (GFP), blood vessels are red (Q-dots), and collagen is blue (secondary harmonic signal).

Movie S3 24-min capture of pinna with NOD scid islets transplanted into a NOD-hCD2-GFP recipient. T cells are green (GFP), blood vessels are white (Q-dots), and collagen is blue (secondary harmonic signal), while dead cells become red through uptake of Sytox dye.

Movie S4 13-min capture of pinna with NOD scid islets transplanted into a NOD-Foxp3-GFP recipient. Foxp3+ T cells are green (GFP), blood vessels are red (Q-dots), and collagen is blue (secondary harmonic signal).

Movie S5 15-min capture of pinna with NOD scid islets transplanted into a NOD-hCD2-GFP recipient (depicted in Figure S1 in Supplementary Material) before injection of antibody. T cells are green (GFP), blood vessels are red (Q-dots), and collagen is blue (secondary harmonic signal).

Movie S6 15-min capture of pinna with NOD scid islets transplanted into a NOD-hCD2-GFP recipient (depicted in Figure S1 in Supplementary Material) after injection of isotype control antibody. T cells are green (GFP), blood vessels are red (Q-dots), and collagen is blue (secondary harmonic signal).

Movie S7 29-min capture of pinna with NOD scid islets transplanted into a NOD-hCD2-GFP recipient (depicted in Figure S1 in Supplementary Material) after injection of agly anti-CD3 antibody. T cells are green (GFP), blood vessels are red (Q-dots), and collagen is blue (secondary harmonic signal).

Movie S8 29-min capture of pinna with NOD scid islets transplanted into a NOD-hCD2-GFP recipient before injection of agly anti-CD3 antibody. T cells are green (GFP), blood vessels are red (Q-dots), and collagen is blue (secondary harmonic signal).

Movie S9 32-min capture of pinna with NOD scid islets transplanted into a NOD-hCD2-GFP recipient after injection of agly anti-CD3 antibody. T cells are green (GFP), blood vessels are red (Q-dots), and collagen is blue (secondary harmonic signal).

Movie S10 59-min capture of a pancreatic islet with immune cell infiltrate in a NOD-hCD2-GFP mouse before injection of antibody. T cells are green (GFP), blood vessels are red (Q-dots), and collagen is blue (secondary harmonic signal).

Movie S11 59-min capture of a pancreatic islet with immune cell infiltrate in a NOD-hCD2-GFP mouse after injection of agly anti-CD3 antibody. T cells are green (GFP), blood vessels are red (Q-dots), and collagen is blue (secondary harmonic signal).
