# Supplementary material for: Non-Invasive Multiphoton Imaging of Islets Transplanted Into the Pinna of the NOD Mouse Ear Reveals the Immediate Effect of Anti-CD3 Treatment in Autoimmune Diabetes
Source: Front Immunol. 2018 May 18;9:1006. doi: 10.3389/fimmu.2018.01006 (PMC5968092; doi:10.3389/fimmu.2018.01006)
Supplement: Supplementary file 12 [file data_sheet_1.PDF]

Supplementary figure 1

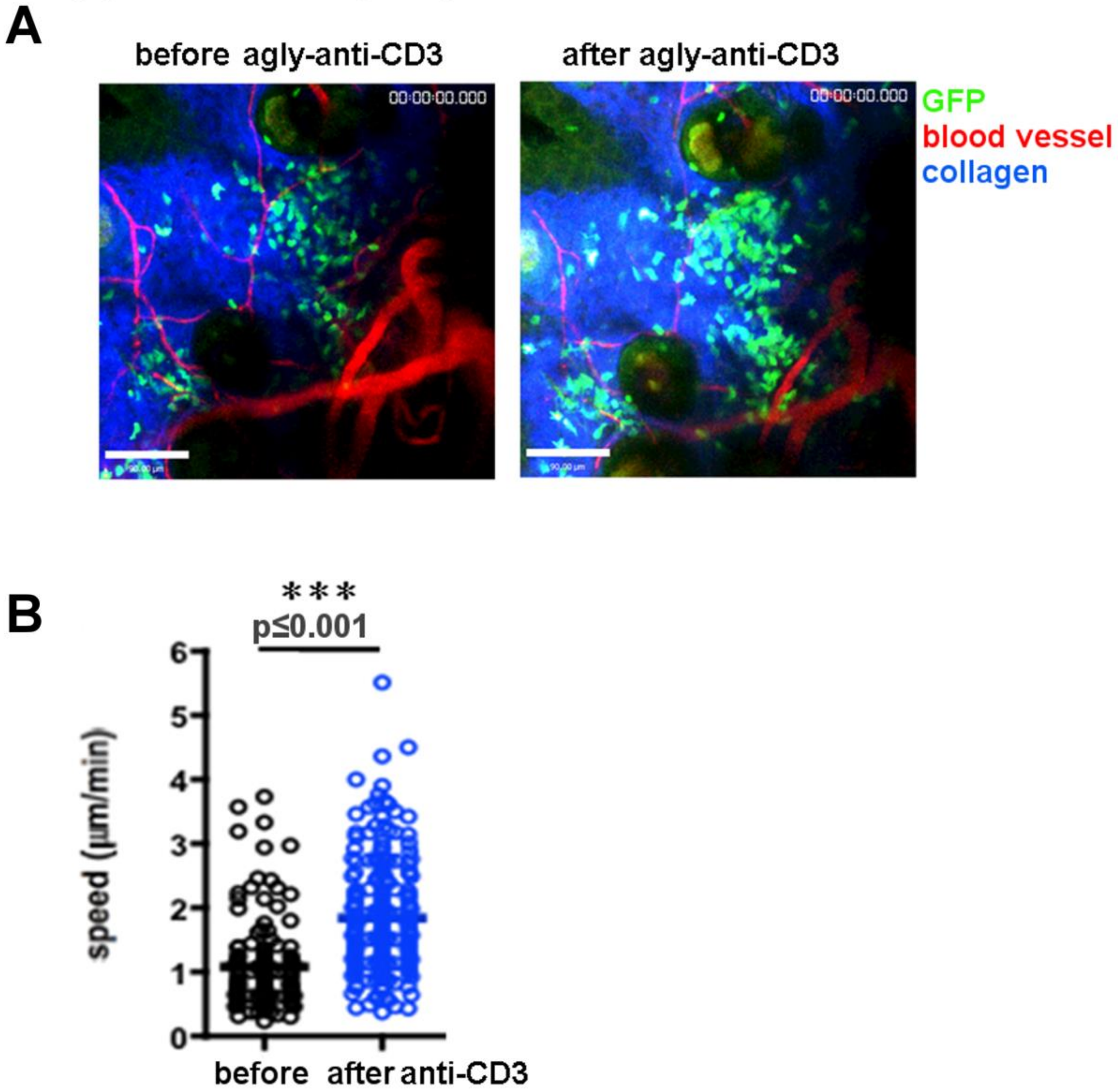

Supplementary figure 1. Imaging of T cell infiltration in NOD-*scid* islets transplanted into the pinna of the ear of a CD2-GFP NOD recipient before and after administration of agly-anti-CD3 performed at the University of Glasgow. (A) On day 0 islets were isolated from a NOD-*scid* mouse and grafted into the pinna of a CD2-GFP NOD mouse. On day 7 the graft recipient was anaesthetized and the graft imaged using multiphoton microscopy. Then 20 µg of agly-anti-CD3 was administered i.v. and the graft imaged again, within 5 minutes of injection. (B) Still images from acquired Z-stack longitudinal multiphoton imaging showing islet-infiltrating GFP^{+} T cells (green) moving around a grafted islet in a 12 week old NOD recipient before injection of aglycosyl anti-CD3 (B, left panel), and after (B, right panel). The white bar represents 90 µm. Red = blood vessels, blue = collagen (secondary harmonic signal), green = GFP. (C) Plotting of the recorded movement of islet infiltrating cells before and after administration of aglycosyl anti-CD3. The difference between time points was assessed using a non-parametric Mann Whitney test, and the data is representative of three independent experiments.

Supplementary figure 2

A

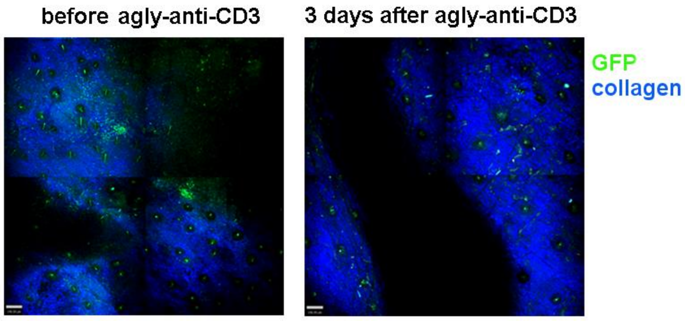

B

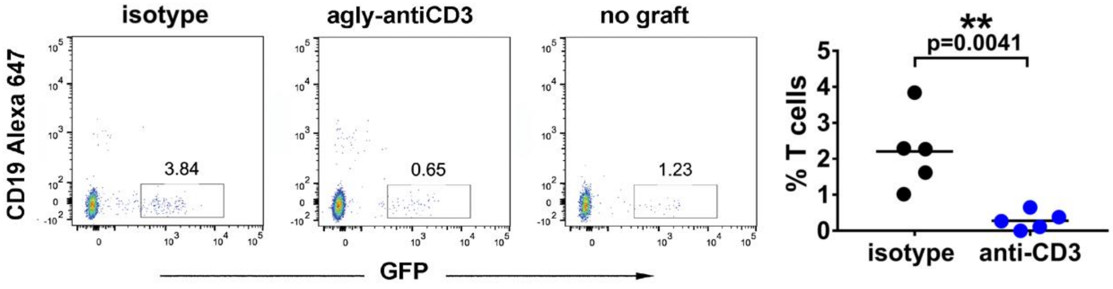

Supplementary figure 2. Administration of agly-anti-CD3 antibody clears infiltrates from islets in the pinna (A) Tile scans of the ventral side of the grafted pinna before (A, left panel) and after three daily injections of 20µg of aglycosyl anti-CD3 on day 7, 8 and 9 after transplant (A, right panel). (B) Flow cytometry based assessment of presence of GFP^+ T cells gated on the $7AAD^-$ population as plotted against $CD19^+$ B cells in pinna islet grafts in mice receiving isotype control or aglycosyl anti-CD3 treatment day 7, 8 and 9 after islet transplant into the pinna (B, left panels), and a graph showing all the recipients in each group, pooled from two experiments, with the difference assessed using a non-parametric Mann Whitney test (B, right panel).
